# The Potential Role of Exosomes in Child and Adolescent Obesity

**DOI:** 10.3390/children8030196

**Published:** 2021-03-06

**Authors:** Ioanna Maligianni, Christos Yapijakis, Flora Bacopoulou, George Chrousos

**Affiliations:** 1 Unit of Orofacial Genetics, First Department of Pediatrics, School of Medicine, National and Kapodistrian University of Athens, “Aghia Sophia” Children’s Hospital, 1 Thivon Street, Goudi, 11527 Athens, Greece; ioanna.malig@gmail.com; 2 University Research Institute of Maternal and Child Health and Precision Medicine, UNESCO Chair on Adolescent Health Care, National and Kapodistrian University of Athens, 8 Livadias Street, Goudi, 11527 Athens, Greece; fbacopoulou@med.uoa.gr (F.B.); chrousge@med.uoa.gr (G.C.); 3 Center for Adolescent Medicine, UNESCO Chair on Adolescent Health Care, First Department of Pediatrics, School of Medicine, National and Kapodistrian University of Athens, “Aghia Sophia” Children’s Hospital, 1 Thivon Street, Goudi, 11527 Athens, Greece

**Keywords:** childhood obesity, adolescent obesity, exosomes, microRNAs, long non-coding RNAs, adipose tissue, epigenetics

## Abstract

Child and adolescent obesity constitute one of the greatest contemporary public health menaces. The enduring disproportion between calorie intake and energy consumption, determined by a complex interaction of genetic, epigenetic, and environmental factors, finally leads to the development of overweight and obesity. Child and adolescent overweight/obesity promotes smoldering systemic inflammation (“para-inflammation”) and increases the likelihood of later metabolic and cardiovascular complications, including metabolic syndrome and its components, which progressively deteriorate during adulthood. Exosomes are endosome-derived extracellular vesicles that are secreted by a variety of cells, are naturally taken-up by target cells, and may be involved in many physiological and pathological processes. Over the last decade, intensive research has been conducted regarding the special role of exosomes and the non-coding (nc) RNAs they contain (primarily micro (mi) RNAs, long (l) non-coding RNAs, messenger (m) RNAs and other molecules) in inter-cellular communications. Through their action as communication mediators, exosomes may contribute to the pathogenesis of obesity and associated disorders. There is increasing evidence that exosomal miRNAs and lncRNAs are involved in pivotal processes of adipocyte biology and that, possibly, play important roles in gene regulation linked to human obesity. This review aims to improve our understanding of the roles of exosomes and their cargo in the development of obesity and related metabolic and inflammatory disorders. We examined their potential roles in adipose tissue physiology and reviewed the scarce data regarding the altered patterns of circulating miRNAs and lncRNAs observed in obese children and adolescents, compared them to the equivalent, more abundant existing findings of adult studies, and speculated on their proposed mechanisms of action. Exosomal miRNAs and lncRNAs could be applied as cardiometabolic risk biomarkers, useful in the early diagnosis and prevention of obesity. Furthermore, the targeting of crucial circulating exosomal cargo to tissues involved in the pathogenesis and maintenance of obesity could provide a novel therapeutic approach to this devastating and management-resistant pandemic.

## 1. Introduction

As defined by the World Health Organization (WHO), overweight and obesity are characterized by potentially detrimental excessive fat accumulation. Since 1975, worldwide obesity has almost tripled, while in 2016, more than 25% of the global population was overweight or obese, including 1.9 billion adults, with over 650 million of them being frankly obese [1]. Despite proposed dietary interventions and fitness programs, and the raised awareness of the obesity epidemic, the prevalence of obesity keeps rising. This incremental tendency could be attributed to the contemporary “obesogenic” environment, which combines physical inactivity with a nutritional transition to high calorie diets and processed foods, but also because of the presence of biological systems preserved through evolution under the pressure of chronic food scarcity [2]. Strikingly, the incremental tendency of obesity seems to include children as well; in 2019, 38 million children under the age of five were overweight or obese [1]. Obesity in childhood and adolescence has been associated with a greater incidence of cardiometabolic comorbidities during adulthood [3], rendering obesity a global leading risk factor of morbidity and mortality. Along with the promotion of a healthy lifestyle, a greater understanding of the functions of adipose tissue and the pathophysiology of obesity constitutes an imperative need for physicians and scientists to confront this modern major threat.

Basic and applied research on obesity has been extensive lately, and mounting evidence has emerged from the promising field of extracellular vesicles (EVs) and their cargo. EVs are surrounded by a lipid bi-layer membrane and may be subdivided size-wise into exosomes (30–120 nm), microvesicles (100 nm–1 μm) and apoptotic bodies (2–3 μm). The latter originate from the blebbing of dying cells and have the size of platelets [4]. Different types of cells release EVs into the extracellular space and/or the blood; these vesicles are capable of transporting their special cargo between nearby or distant cells, mediating intercellular communication [5]. Their cargo comprises a variety of molecules, including proteins, DNA and RNA (mRNAs and non-coding RNAs) and is determined by the type and metabolic and nutritional state of the originating cells. The presumptive involvement of EVs in the etiology of obesity has not been extensively studied.

Adipose tissue, apart from being an energy storage depot, acts also as an endocrine gland, secreting many bioactive peptides (adipokines) and metabolites that travel to distant cells and tissues and influence their function. Interestingly, adipocytes also secrete exosomes that can modulate the activity of cells within or outside adipose tissue in an autocrine, paracrine or endocrine manner. Thus, preadipocytes, endothelial cells, immune cells and other cells within adipose tissue or other distant tissues may be affected by exosomes [6]. Exosomes and their cargo are altered in obesity [7]. Furthermore, exosomal microRNAs (miRNAs) appear to be involved in obesity and its cardiometabolic sequelae by acting as signaling entities, mediating the crosstalk among adipose tissue, liver, skeletal muscles and immune cells, and promoting inflammation in these organs [8]. These biological properties of exosomes and the non-coding RNAs they contain suggest that they could hold great potential for the diagnosis and treatment of obesity and its detrimental complications.

The scope of this review includes the role of exosomes and their cargo in adipose tissue physiology, as well as their probable dysregulation and contribution in the multifactorial obese state in children and adolescents, incorporating data from human clinical studies and animal models. Therefore, we systematically researched PubMed using various combinations of the subsequent words: “exosomes,” “obesity,” “children,” “adolescents,” “miRNAs,” “lncRNAs,” “adipose tissue,” “adipogenesis,” “epigenetics” and “stress”. The most recent, complete and relevant reviews and original articles on the topic, published up to November 2020, were selected.

## 2. Exosomes

### 2.1. Biogenesis and Secretion of Exosomes

**Figure 1:**
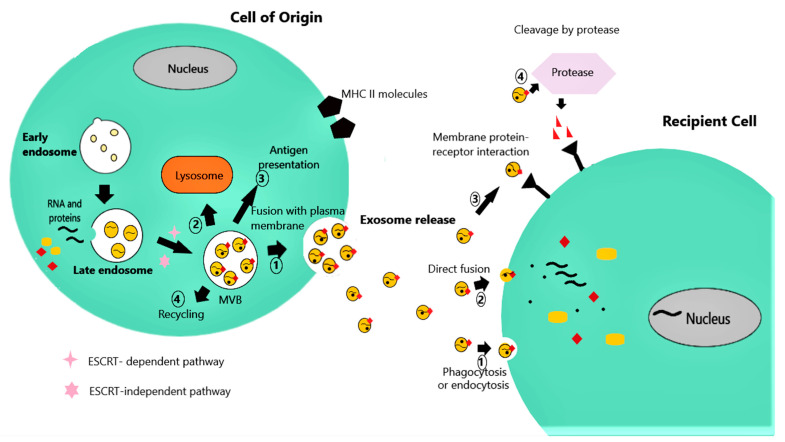
Exosome biogenesis and secretion. Exosomes are generated by reverse invagination of the membrane and incorporation of RNA cargo and proteins during endosome maturation to multivesicular bodies (MVBs). The assembly of exosomes is regulated either by the recruitment of the endosomal sorting complex required for transport endosomal sorting complex required for transport (ESCRT) complex (ESCRT-dependent pathway), or by different proteins such as the tetraspanins, and the lipid ceramide (ESCRT-independent pathway). Subsequently, MVBs can follow several pathways: (1) fusion with the plasma membrane and exosome release; (2) fusion with lysosomes for content degradation; (3) participation in antigen presentation in the plasma membranes by major histocompatibility complex (MHC) class II molecules; and (4) recycling. Upon release, exosomes can interact with the plasma membranes of target cells and trigger downstream effects by (1) phagocytosis or endocytosis; (2) direct fusion of exosome membrane with recipient cell membrane; (3) direct interaction of exosome membrane protein with recipient surface cell receptor; (4) cleavage by proteases of exosome membrane proteins and binding of the resulting fragments to recipient cell receptors.

Exosomes are vesicles enclosed by a lipid membrane and have a diameter ranging from 30 to 120 nm, and an internal structure similar to that of a simple microbial cell [9]. The biogenesis of exosomes begins within the endosomal system (Figure 1). In the course of endosome progression into multivesicular bodies (MVBs), exosomes originally form as intraluminal vesicles (ILVs), created by reverse membrane invagination [10]. One of the major pathways for the biogenesis of exosomes relies on the “endosomal sorting complex required for transport” (ESCRT). The recruitment of the four ESCRT subunits serves as a sorting mechanism of ubiquinated proteins into the vesicles and guides exosome wrapping, finally leading to exosome formation [11]. After depletion of all four ESCRT subunits, the ESCRT-independent pathway, consisting of several proteins such as the tetraspanins (CD9, CD63, CD81, CD82) and the lipid ceramide, regulates the assembly of exosomes. These proteins are abundant in the exosomes and facilitate incorporation of their cargo and membrane curvature [11]. MVBs can subsequently follow different pathways [12]: (a) content degradation by fusion with lysosomes, (b) exosome release into the extracellular space by fusion of the ILVs with the plasma membrane, (c) participation in the cell surface expression major histocompatibility complex (MHC) class II molecules taking part in antigen presentation or (d) recycling (Figure 1).

Various proteins modulate MVB fusion with the plasma membrane and subsequent exosome release. More specifically, Rab GTPases act on exosome traffic and fusion with the cell membrane in a cell-specific manner, meaning that different cells can use distinct Rab proteins to modulate exosome release [13]. Furthermore, another group of proteins, the soluble N-ethylmaleimide-sensitive factor attachment protein receptors (SNARE), play an important role in vesicle tethering and membrane fusion, also influencing exosome release [13]. Exosome biogenesis and secretion is additionally influenced by the cell metabolic status, depending on factors such as ceramide metabolism, endoplasmic reticulum stress (ER stress), autophagy, and intracellular calcium [10]. Interestingly, adiponectin, a protein produced only by adipocytes, triggers exosome generation by endothelial and skeletal muscle cells expressing T-cadherin [14], while exosome secretion from endothelial cells in adipose tissue is regulated by glucagon [15]. In summary, exosome biogenesis and metabolic pathways seem to interact bi-directionally, as the former is metabolically regulated, while the latter is influenced by exosomes.

After release, exosomes interact with their target cells by transferring their bioactive cargo into them, thus, triggering phenotypic changes in the recipient cells (Figure 1). The targeting of specific cells is mediated by multiple exosomal surface molecules, such as phosphatidylserine receptors, lectins, integrins and other cell adhesion molecules, that enable the selective interaction with the corresponding receptors on the plasma or endosome membrane of recipient cells [9]. This interaction may be accomplished through one of the following pathways [12]: (a) exosome phagocytosis or endocytosis or direct exosomal- cell plasma membrane fusion, all delivering exosomal cargo into the recipient cell cytoplasm and activating downstream events, (b) direct activation of recipient cell membrane receptors by exosome membrane proteins, stimulating intracellular signaling cascades without being internalized, and (c) cleavage of exosome membrane proteins by proteases and binding of the resultant soluble fragments to cell surface receptors.

### 2.2. Exosomal miRNAs and lncRNAs

Generally, exosomes play a role in regulating metabolic homeostasis by transporting their specific cargos between different cell types. Exosomes transfer various types of biomolecules, such as proteins, DNA, mRNA and non-coding RNAs (ncRNAs). ncRNAs lack protein-coding capacity and they better reflect life complexity than the protein-coding genes, as they represent the majority of transcripts in the human genome that participate in dynamic gene regulation, mediated by a complex interaction with DNA, other transcripts and/or proteins [16]. ncRNAs are subdivided into long non-coding RNAs (lncRNAs) and short ncRNAs, including microRNAs (miRNAs), according to their nucleotide length.

miRNAs. One exosomal cargo that has attracted particular interest lately for its possible diagnostic and therapeutic applications is miRNAs, a cohort of endogenous ncRNA transcripts with an approximate length of 20–22 nt, that are involved in the regulation of gene expression, primarily post-transcriptionally [17,18]. In general, the mechanism by which miRNAs regulate gene expression is by interacting with complementary base pairs of specific mRNAs, mainly at their 3′-untranslated region (UTR), and/or by down-regulating gene transcription. However, according to recent findings, miRNAs can up-regulate gene-transcription as well, by the formation of triplexes that interact with gene promoter regions, or by acting synergistically with other miRNAs to regulate gene transcription [19]. Circulating miRNAs are secreted into the systemic circulation either inside exosomes and other specific traffic and sorting mechanisms, or by passive leakage from broken cells [20]. miRNAs can be used as biomarkers for many different pathological conditions, and as a potential therapeutic target modulators of various functions [21].

lncRNAs. These have a length of more than 200 nt and bear minimal to no protein coding capacity [22,23], contributing to the initial notion that they represented “junk RNA.” Nevertheless, recent findings have shown that lncRNAs play important roles in regulating physiological processes [24,25], and are dysregulated in a variety of disorders, such as cardiovascular and neurodegenerative diseases and cancers [26]. Contrary to short ncRNAs, lncRNAs are subject to post-transcriptional alterations, including polyadenylation and splicing [23]. Generally, lncRNAs can be classified into intergenic, antisense, divergent, intronic and enhancer lncRNAs, according to their relative localization with neighboring or distant coding genes [16]. Their secondary structure is intricate and thermodynamically stable, enabling their interaction with proteins, DNA, mRNA and other non-coding RNAs, such as miRNAs, by acting as scaffolds for other biomolecules and by facilitating their transport into exosomes [23,27].

lncRNAs influence nuclear and cytoplasmic epigenetic functions (chromatin rearrangement and miRNA sponging), as well as translation and degradation of mRNA, and are involved in orchestrating gene expression at multiple levels [27]. Lately, lncRNAs have been identified within exosomes [28]. Although the mechanism of their loading in exosomes remains to be deciphered, specific RNA structure patterns and protein co-operators in charge of intracellular compartmentalization and setup of exosomal cargo may be contained within lncRNAs. The parent cell type and changes in the cellular environment regulate the distribution of lncRNAs in exosomes [4]. The special characteristics that exosomal lncRNAs possess, make them potential candidate biomarkers for various diseases [27], because (a) lncRNAs inside exosomes are protected from RNases, maintaining their integrity and function, (b) the content of exosomal lncRNAs is higher than in other types of EVs, and (c) the exosomal lncRNA profile is specific for each tissue. The roles of exosomal lncRNAs have only been partly demonstrated; it has been recently suggested that they could transport phenotypic modifications such as drug resistance, promotion of angiogenesis and induction of carcinogenesis to neighboring or distant cells [27].

### 2.3. Function(s) of Exosomes

Intensive research conducted over the last decade has revealed that the functions of exosomes are determined by their originating cells or tissues and reflect their condition at the time of exosome biogenesis [12]. Exosomes are generated and secreted by a variety of cell types, including nervous system cells, epithelial cells, fibroblasts, adipocytes and cells of the immune and hematopoietic systems [4]. Exosomes have been isolated in various biological specimens, including blood, urine, semen, breast milk, saliva, amniotic fluid, cerebrospinal fluid, synovial fluid and bronchoalveolar lavage [4]. Exosomes predominately mediate intercellular communication, having a significant impact both in physiological and pathological biological processes [4]. Exosomes enable remote cells to exchange genetic and epigenetic information, thus allowing a genetic/epigenetic synchronization of cells of the same or different tissues. When released under normal circumstances, exosomes are involved in cell differentiation, maintenance of cellular homeostasis, regeneration of tissues, and the proper functioning of the immune system [9,12]. These processes are mediated not only by the signaling properties of exosomes, but also by their ability to act as a waste removal pathway to lysosomes; misfolded or accumulated harmful proteins and cytoplasmic DNA, can be released in exosomes, thus attenuating cellular stress and maintaining homeostasis [29].

Acute stress exposure may lead to alterations in both exosomal membrane proteins and cargo, and these changes may cause a shift of immunomodulatory properties. Under pathological circumstances, exosomes may potentiate cellular stress causing cell damage [30]. Exosome cargo may also alter the inflammatory responses of recipient cells [31]. A number of cytokines predisposing to inflammation, such as interleukin-1 beta (IL-1β), interleukin-6 (IL-6), and tumor necrosis factor-alpha (TNF-α), as well as other inflammation-associated molecules like heat-shock proteins, have been associated with exosomes [32]. Also, there is evidence of involvement of exosomes in a broad spectrum of diseases, including cancer, heart failure, and liver and neurodegenerative diseases [30]. Moreover, exosomes play an important role in viral infections; virus-infected cells secrete exosomes containing a variety of cellular products of the host and the virus, capable of modifying the host cellular responses [33]. Considering that exosome-mediated intra-adipose and inter-organ communication appears to be of great significance for energy metabolism, there seems to be a special function of exosomes in adipose tissue biology, which may be disrupted in obesity.

## 3. Adipose Tissue Physiology

### 3.1. Adipose Tissue Depots

Adipose tissue is a functionally pleiotropic tissue, considered to be of mesodermal origin [34,35]. Adipogenesis is defined as precursor cell differentiation into mature adipocytes, committed to fat storage and regulation of energy balance, and is influenced by numerous factors, such as endocrine and steroid hormones, cytokines, miRNAs, cytoskeletal proteins and transcription factors, among others [35]. The transcription factor peroxisome proliferator activated receptor gamma (PPARγ) is regarded as the master regulator of adipogenesis [35]. Adipose tissue is traditionally subdivided into white adipose tissue (WAT) and brown adipose tissue (BAT), possessing different developmental origins, morphology, metabolic functions and gene expression patterns. Lately, the existence of another type of adipose tissue, “beige” adipose tissue, has also been proposed.

WAT. This tissue constitutes the main site of energy storage [36]. After a meal, white adipocytes, the main cell type of WAT, under the guidance of insulin, take up fatty acids released from circulating triacylglycerols (TAGs) via the action of lipoprotein lipase and store them in the form of triglycerides in a large central lipid droplet. Conversely, during fasting and in other catabolic situations, WAT delivers free fatty acids into the circulation to supply the necessary energy for the function of other tissues and organs [34]. However, when caloric intake exceeds energy expenditure, WAT cells increase in size (hypertrophy), as well as in number (hyperplasia). Hyperplasia is believed to correlate with a healthier pattern of AT expansion, while hypertrophied adipocytes are characterized by an aberrant secretion of adipokines [19]. These include monocyte chemoattractant protein 1 (MCP-1), IL-6 and/or TNF-α, which promote macrophage recruitment and enduring inflammation, and disrupt normal adipocyte metabolism [19]. Long-lasting cellular overflow of nutrients and metabolites and the ensuing hypoxia of hypertrophic adipocytes may overwhelm homeostatic protective mechanisms and trigger cell stress responses, leading even to cell apoptosis [2].

Obesity, especially the early-onset childhood type, is characterized by increased numbers of preadipocyte precursors and adipocytes with an impaired metabolism and reduced proliferative potential [37]. As obesity worsens, many metabolic processes become dysregulated, arising from increased blood-stream free fatty acids (FFA), increasing local inflammation, and related to a switch of adipose-tissue macrophages (ATMs) to a pro-inflammatory M1 phenotype, ultimately causing reduced responsiveness of adipocytes to insulin, that is, insulin resistance [38]. Adipocyte insulin resistance disrupts the storage of lipids in WAT and causes aberrant lipid accumulation in the liver and skeletal muscles, thus contributing to generalized insulin resistance and type 2 diabetes mellitus (T2DM) [39].

Metabolically unhealthy obesity is dominated by intra- and extra-abdominal WAT characterized by insulin resistance and inflammation and correlates more to visceral abdominal WAT (vWAT) than to subcutaneous WAT (sWAT) [36]. According to transcriptome studies, genes associated with adipocyte metabolism, adipokine secretion and cell signaling display a different pattern of expression between sWAT and vWAT [40], while pathway analysis has shown that genes implicated in inflammation are better expressed in vWAT than sWAT. The reason for these differences has not been fully elucidated but could be partly attributed to the passage of visceral fat products through the portal vein and the resultant drainage into the liver circulation, ultimately leading to systemic metabolic disease and low-grade inflammation [36].

BAT. This is a richly vascularized tissue that correlates negatively with risk of metabolic disease and the body-mass index (BMI) [41]. It functions by dissipating energy in the form of heat, via the catabolism of lipids and carbohydrates, in a diet- or cold-induced procedure referred to as “adaptive or non-shivering thermogenesis.” Contrary to white adipocytes, brown adipocytes contain microscopic lipid droplets and a significantly higher amount of mitochondria containing uncoupling protein 1 (UCP1), a transmembrane protein which contributes to heat production by creating a proton leak and decoupling the electron transfer chain from ATP biosynthesis [41]. During the first decade of life, BAT is widespread throughout the body, while in infancy the main thermogenic depot is interscapular BAT. Moreover, BAT amount and activity (as measured by positron emission tomography-computed tomography (PET-CT)) rises during adolescence and is closely associated to muscle gain, as well as body weight and adiposity alterations, with obese children having markedly less BAT in the supraclavicular area than lean children [42]. As age advances and body weight increases, the amount of BAT is typically reduced, indicating a probable role in pathologies related to aging and obesity [42]. A transgenic mouse overexpressing UCP1 has resistance to obesity development and polymorphisms in UCP genes in children have been associated with obesity [43]. Of note, inflammatory responses also occur in BAT, including macrophage recruitment and secretion of proinflammatory cytokines, hampering brown adipocyte proliferation and differentiation capacity, and promoting apoptosis, finally reducing the thermogenic activity of BAT [44]. In children and adolescents, the preservation of their inherently increased BAT amount and activity, and the inhibition of the declining tendency of BAT with advancing age, could potentially represent a novel therapeutic approach to obesity.

Beige AT. It has been observed that following various stimuli, including cold exposure, beta-adrenergic stimulation, diet, exercise, as well as adipokines, adipocytes referred to as “beige”, that resemble brown adipocytes, arise within WAT and gain thermoregulatory functions, in a process called “WAT browning” or “beiging” [35]. Even though beige adipocytes significantly resemble brown adipocytes functionally in features such as increased UCP1 expression levels, it has been indicated that beige adipocytes constitute a distinguishable subset of adipocytes, arising either from a distinct precursor or from transdifferentiation of white adipocytes [35]. WAT “browning” could represent a novel way to combat obesity and related metabolic complications by compensating for the reduction of BAT activity with advancing age and increasing body weight.

### 3.2. Endocrine Properties of Adipose Tissue

As previously mentioned, apart from substantially regulating fatty acid metabolism, both WAT and BAT produce and secrete many adipokines, as well as regulatory lipids, called lipokines, that transduce signals in an autocrine, paracrine or endocrine manner [34,45]. Among them, leptin (LEP), a peptide hormone mainly released from WAT, mediates a complex communication between the intestine, adipose tissue and the brain, leading to reduced food consumption and enhanced energy expenditure, thus regulating body weight homeostasis [46]. Even though the increase of adipose tissue mass in obesity increases production of leptin, soluble leptin receptors (LEPRs, the main leptin binding proteins in circulation that result from LEPR cleavage and regulate leptin function and bioavailability) decrease, while leptin transport into the central nervous system (CNS) is not commensurately increased, resulting in decreased hypothalamic leptin signaling, increased leptin levels and leptin resistance [47,48,49,50]. Adiponectin, another adipocyte-specific hormone, actually the most abundant adipocytokine in plasma and ubiquitously expressed in adipose tissue [10], displays anti-inflammatory and antifibrotic properties in many organs [51,52,53]. By still undefined exact mechanisms, obesity and obesity-related cardiometabolic disease are characterized by low plasma adiponectin levels [34,54]. Intriguingly, as already mentioned, there is evidence that adiponectin stimulates exosome biogenesis and secretion, and that T-cadherin, which is an adiponectin receptor, promotes adiponectin aggregation in MVBs, thus regulating adipose tissue exosomal secretion and possibly communication with other tissues [14]. The large number of adipokines and lipokines has been more comprehensively reviewed elsewhere [34].

### 3.3. Adipose-Derived Exosomes

Lately, the up-and-coming research field of adipose-derived exosomes has been attracting interest as a novel type of complex biomolecules mediating inter-cellular and inter-organ communication. The adipose tissue origin of exosomes is ascertained by the identification of adipocyte-specific protein markers, such as fatty acid binding protein 4 (FABP4), adiponectin, and perilipin A [55]. Accumulating evidence supports the hypothesis that adipose tissue secretes exosomes that enter the blood circulation and, thus, orchestrate systemic carbohydrate homeostasis in an endocrine fashion [56]. Adipose-derived exosomes are secreted by adipocytes, as well as other cells residing within AT, such as ATMs, into the systemic circulation to travel to their target cells/organs. For example, adipose-derived exosomes seem to mediate communication between adipocytes and endothelial cells within and outside adipose tissue [15].

It has been proposed that exosomes regulate metabolic processes by also modifying immune function. In one study, adipocyte-derived exosomes promoted monocyte differentiation into active macrophages and elevated levels of IL-1β and TNF-α, well-known pro-inflammatory cytokines [6], while in another study, this pro-inflammatory effect was more prominent for vWAT-released exosomes of adipocyte origin [57]. Conversely, ATMs secrete exosomes that interfere with the inflammatory state and metabolic status of adipose tissue [58]. However, adipose-derived exosomes also have anti-inflammatory properties; thus, adipose-derived stem cells (ADSC) released exosomes that triggered an anti-inflammatory M2 polarization of ATMs by transporting signal transducer and activator of transcription 3 (STAT3), which transactivated arginase-1 [59]. Generally, it has been proposed that adipose-derived exosomes exert their metabolic actions by transporting their specific cargos to other cells. Below, we review the existing evidence about the roles of exosomal and circulating miRNAs and lncRNAs in adipocyte biology.

microRNAs in adipocytes. Adipose-derived miRNAs represent a big part of circulating miRNAs and are mainly contained in exosomes secreted both from WAT and BAT [7]. They play a significant role in the inter-cellular and inter-organ communication required to maintain energy homeostasis [7,60] and their distinct expression profile and function is determined by the degree of excess adiposity [61]. Furthermore, ATMs secrete exosomal miRNAs, which regulate adipocyte functions by regulating systemic glucose metabolism [58]. Many circulating miRNAs were downregulated in patients suffering from lipodystrophy, suggesting the contribution of adipose tissue in circulating miRNAs in humans [7]. Importantly, a mouse model specifically lacking Dicer in adipose tissue (ADicerKO mice) has been extensively investigated and has helped uncovering new aspects of the role of adipose-derived miRNAs in regulating biological processes. Dicer is an enzyme that catalyzes miRNA processing; ADicerKO mice have impaired miRNA processing, especially in adipose tissue, and reduced levels of circulating miRNAs, as well as reduced WAT mass, whitening of BAT, AT inflammation, insulin resistance and dyslipidemia [7]. Interestingly, the levels of circulating miRNAs recovered and glucose tolerance was ameliorated in ADicerKO mice after transplantation of wild-type mouse-derived adipose tissue [7]. These findings suggest that adipose-derived miRNAs constitute a significant proportion of circulating miRNAs and that they may have key roles in regulating systemic energy metabolism.

Specifically, transplantation-induced improvement of glucose tolerance in ADicerKO mice correlated with a lower level of hepatic and systemic fibroblast growth-factor 21 (FGF21) [7]. BAT-derived exosomal miR-99b maintained glucose normal homeostasis and regulated whole-body metabolism by suppressing hepatic production of fibroblast growth factor-21 (FGF-21) [7], thus, suggesting a major mechanism of adipose tissue-liver communication by BAT-derived exosomes. However, these findings contradict the notion that FGF21 has anti-obesity and anti-diabetic properties [62]. Further experimental evidence is needed to define the exact mechanism of miR-99b and FGF-21 action in energy metabolism. Another BAT-derived exosomal miRNA, miR-92a, inversely correlated with human BAT activity in healthy subjects, as well as in WAT of mice upon cold exposure, suggesting its potential use as a biomarker for BAT activity [63].

Lately, the role of circulating miRNA in adipocyte biology has attracted researchers’ interest. At WAT, miRNAs have been implicated in adipogenesis, endocrine function of AT, glucose and lipid metabolism and WAT hypoxia and browning [19]. A group of miRNAs have been correlated with promotion of adipogenesis through various mechanisms. Among other mechanisms, miR-143 enhances adipocyte differentiation via the mitogen-activated protein kinase (MAPK) signaling pathway [64], while miR-21 regulates the transforming growth factor beta (TGF-β) pathway in adipose-derived mesenchymal stem cells (MSC) [65]. Conversely, other miRNAs, such as miR-130, inhibit adipocyte differentiation by suppressing PPARγ activity [66]. Furthermore, in WAT inflammation, several miRNAs modulate adipokine production, such as MCP-1, TNF-α and adiponectin [67]. Regarding WAT browning, specific miRNAs regulate brown adipocyte transcriptional modulators, such as miR-30. MiR-455 enhances mitochondrial biogenesis by activation of AMP-activated protein kinase 1a (AMPK1a) [19,68]. Interestingly, cold exposure stimulated WAT browning in adipose-specific miR-455 transgenic mice [19].

lncRNAs in adipocytes. Several studies aiming to construct a lncRNA database in adipose tissue have proposed various lncRNAs as significant regulators of adipocyte biology, white adipocyte differentiation and function, and brown and beige adipose tissue thermogenesis [69]. Recently, 175 circulating lncRNAs were reported to be specifically regulated during adipogenesis, using whole transcriptomic profile of undifferentiated and mature adipocytes derived from WAT and BAT [70]. More specifically, lncRNA steroid receptor RNA activator (SRA) was demonstrated to promote adipocyte generation and function via various mechanisms, including insulin-related signal transduction [23]. Correspondingly, mice with SRA knockout displayed resistance to obesity caused by a high-fat diet (HFD), decreased adipose tissue mass and gene expression of adipocytes, reduced plasma TNFα, and enhanced insulin sensitivity [71]. Apart from SRA, lncRNAs regulated in adipogenesis (lnc-RAPs), Plnc1, super-long intergenic nc RNA functioning in adipocyte differentiation (slincRAD), PU.1 antisense (AS), HOX antisense intergenic RNA (HOTAIR), adipogenic differentiation induced nc RNA (ADINR) and nuclear enriched abundant transcript 1 (NEAT1) were involved in white preadipocyte differentiation mainly through modulation of key adipogenic molecules, such as PPAR-γ and CCAAT-enhancer-binding protein (C/EBP) [23]. Interestingly, NEAT1 interacted with miR-140, thus taking part in miR-140-induced adipogenesis. MiR-140 knockout downregulated NEAT1 and remarkably decreased expression of PPARγ and C/EBP and fat accumulation, effects recovered after re-expression of NEAT1 [72].

In BAT, both brown fat lncRNA 1 (Blnc1) and lncRNA BAT enriched (lncBATE-1) induced brown and beige adipogenesis through the formation of a ribonucleoprotein complex with key transcriptional factors of thermogenesis [69]. Brown preadipocytes with lncBATE1 blockade had attenuated the expression of brown fat and mitochondrial markers [69]. Brown and beige adipogenesis, as well as thermogenic stimuli, induce Blnc1, which interacts with transcription factor early B-cell factor 2 (EBF2) and stimulates thermogenic genes. Furthermore, Blnc1 was proposed to decrease pro-inflammatory signaling and obesity-induced inflammation of AT, along with improving insulin sensitivity [23]. In a recent study, lncRNAH19 was related to brown adipocyte differentiation and was inversely correlated with human BMI, while H19 transgenic mice were protected from obesity caused by a HFD, probably because of improved mitochondrial biogenesis and fuel oxidation in WAT and BAT [73]. To date, several circulating lncRNAs appear to regulate adipocyte biology, however, more studies will increase our understanding of adipocyte function and their contribution to human obesity.

## 4. Child and Adolescent Obesity

### 4.1. Current Knowledge

Growth charts developed by the Centers for Disease Control and Prevention (CDC), based on data from national surveys, are used for the classification of overweight and obese status in children and adolescents, according to the age- and gender-specific percentiles of body mass index (BMI). Overweight is indicated by measurements over the 85th percentile and under the 95th percentile on age- and gender-specific body curves, while obesity is indicated by measurements over the 95th percentile [74]. A BMI equal to or greater than the 120% of the 95th percentile defines severe obesity in children and adolescents [74].

It is predicted that children and adolescents defined as overweight or obese will have a progressively higher BMI during adulthood [74]. Similarly to adults, all crucial organ systems are affected in adolescent obesity, often contributing to a higher morbidity by promoting inflammation and increasing the risk of the so-called “chronic noncommunicable disorders” in adulthood. These include the metabolic syndrome and its components, including hypertension, dyslipidemia, endothelial dysfunction, insulin resistance, T2DM, and nonalcoholic fatty liver disease (NAFLD), as well as cardiovascular disease [3,75]. Obesity and its related disorders are largely preventable, rendering primary prevention, timely diagnosis and secondary prevention a high priority [1].

Commonly, obesity is primarily caused when endogenous and exogenous factors result in a discrepancy between energy intake and energy consumption, finally leading to excess weight gain. These include genetic and epigenetic, dietary, physical, psychosocial, and other environmental factors, which could all impact negatively on overall energy homeostasis [76]. Furthermore, obesity could accompany other diseases, such as various genetic obesity syndromes, disorders of the endocrine and nervous system associated with specific clinical features and frequently delayed growth [77,78,79]. To better understand the complex obese state, we review briefly evolutionary/genetic and developmental/epigenetic contributing factors, focusing on the latest data concerning the possible involvement of exosomes and their cargo.

### 4.2. Evolutionary and Genetic Basis of Obesity

Genetic types of obesity present with a common clinical spectrum and can be classified into mendelian (monogenic) syndromic obesity, mendelian non-syndromic obesity, and polygenic obesity, which is most common [80]. Monogenic obesity attributed to relatively rare genetic abnormalities and pathogenic variations of genes encoding key proteins regulating energy homeostasis, may be combined with dysmorphic features, mental retardation and organ-specific congenital anomalies. Two well-known obesity syndromes are those called Prader-Willi and Bardet-Biedl [81,82]. Currently described non-syndromic forms of mendelian obesity comprise mainly genetic defects in the leptin/melanocortin pathway, such as mutations in the genes encoding LEP, LEPR, prohormone convertase 1 (PC1/3), proopiomelanocortin (POMC) and melanocortin receptor 4 (MC4R), leading to overeating [79,83]. Conversely, in polygenic obesity types, various frequent genetic variations, each one having a limited effect, act in a cumulative way [84]. For example, common variations in intron 1 of the fat mass and obesity-associated (FTO) gene contribute to polygenic obesity and account for about 1–2% of BMI deviation in the general population.

It has been postulated that the difficulty encountered by many people in their attempt to lose weight and maintain weight loss could be attributed to powerful biologic systems protecting our body weight during our evolution, thus ensuring survival and adequate reproduction in a food-deficient environment, but contributing to obesity development in an obesogenic environment where food is abundant [2]. The combination of various gene polymorphisms accounts for the subtle individual differences in metabolism and the heterogeneity of the obesity phenotype regarding onset and severity, depot- and gender-specific fat accumulation, as well as success of body weight loss attempts.

### 4.3. Environmental and Epigenetic Contributing Factors

Environmental contributors to obesity are complex and deep-rooted in modern society. These include a recent shift to plentiful, highly palatable processed food in combination with the adoption of a sedentary lifestyle and a disruption in normal circadian rhythms [2]. Furthermore, it has been revealed that chronic stress plays a major role in the development of obesity and its complications [85,86]. Chronic hypersecretion of stress mediators, such as glucocorticoids, may result in insulin and leptin hypersecretion, contributing to insulin and leptin resistance, along with dysregulation of appetite and food intake by inducing alterations in the reward system, finally leading to obesity [86]. Also, overexpression of 11β-hydroxysteroid dehydrogenase type 1 (11β-HSD1) resulting in increased conversion to active cortisol, has been associated with central obesity because of increased activity in the adipose tissue of obese adult females [48]. Importantly, the offspring of obese mothers display a higher baseline cortisol, resulting in a dysregulated stress response and predisposition to metabolic dysfunction [48].

Epigenetic contribution to obesity has attracted great interest, as it helps understand the interaction between genes and environment. There are rapid, dynamic and reversible or long-term less reversible adaptive alterations of genome activity without concurrent changes in the DNA sequence, following environmental stimuli [87]. Environmental factors, including nutrition, physical activity, lifestyle, sleep habits, chronic stress, pollutants and so forth, can trigger the onset of obesity by epigenetically modifying genes involved in multiple biological processes in metabolism and immunity [48]. Epigenetic changes may occur during fetal life, early childhood, and adolescence and to a lesser degree, in adulthood, and encompass DNA methylation, glycosylation, myristoylation, post-translational histone modifications, as well as nc RNAs orchestrating a variety of cellular processes, including gene expression [88].

Significantly, maternal under- or over-nutrition, as well as maternal insulin resistance and weight gain during pregnancy are markedly associated with offspring obesity [2]. Even though the existing evidence mainly attributes these effects to DNA methylation and histone modifications, recent animal findings unveil miRNA alterations caused by an unfavorable intrauterine environment as potential contributors to offspring obesity. For instance, in rats, maternal protein restriction led to the programmed increase of imprinted miR-483 in offspring epididymal WAT (eWAT). This resulted in decreased expression of growth differentiation factor 3 (GDF-3) and subsequent inhibition of adipogenesis and ectopic fat accumulation [89]. Interestingly, in AT from low birthweight humans miR-483 was increased, with a concomitant decrease in GDF-3 [89]. On the other hand, maternal HFD induced a programmed increase in miR-126, resulting in reduced insulin receptor substrate-1 (IRS-1) in eWAT of male offspring [89]. Furthermore, apart from maternal contribution, there is mounting evidence for the intergenerational transmission of paternal metabolic disease risk via EVs. Environmental adverse factors are imprinted in the specific EVs cargo and transferred to sperm [90]. Intriguingly, sperm from HFD-fed males contains miRNAs that cause offspring obesity when transported into embryos [88]. Below, we focus on the special contribution of nc RNAs (miRNAs and lncRNAs), identified within exosomes or directly in blood circulation, in obesity development and maintenance.

Exosomal miRNAs in obesity. Obesity has been associated with increased circulating EVs, including exosomes [55]. The pro- or anti-inflammatory properties of exosomes and their ability to act as mediators of communication among the liver, AT, skeletal muscles and the immune system, may account for their roles in obesity and obesity-associated inflammation [8]. These inflammation-related properties of exosomes may be accomplished via their unique cargos, especially miRNAs, as demonstrated by the reduced metabolic effects of miRNA-depleted exosomes [58]. A growing body of evidence implicates exosomal miRNAs, such as miR-155 and miR-27a, in the crosstalk between adipocytes and ATMs, which is an important determinant in obesity development [91]. More specifically, miR-155 is increased in adipocyte-derived exosomes in obese mice as compared to lean mice and it can stimulate proinflammatory M1 macrophage polarization and impair BAT activity by inhibiting C/EBP [58]. MiR-27a displays similar activity in macrophages and represses adipocyte differentiation, while increased serum levels of this miRNA are related to obesity in children [91]. Adipose-derived exosomal miRNAs, whose profile is altered in obese female teenagers, alter Wnt/β-catenin and TGF-β signaling, which regulate adipogenesis and inflammatory responses [60]. Moreover, the dysregulated profile of circulating exosomal miRNAs is modified one year after gastric bypass surgery in adults [91]. Lastly, it has been proposed that microbes excrete miRNA-containing vesicles, which could potentially contribute to the metabolic impact of gut microbiota dysbiosis on obesity and related complications [92].

Circulating miRNAs in child and adolescent obesity. Up to now, most studies have been conducted by identifying circulating miRNAs in blood samples rather than exosomal or even adipose-derived exosomal miRNAs and have been mostly focused in adult obesity, with a general scarcity of data regarding miRNA involvement in childhood obesity [93]. However, some recent studies have evaluated circulating miRNAs in blood samples in several childhood disorders, such as diabetes and obesity [94,95,96]. Obese children overexpressed four particular circulating miRNAs (miR-222, miR-142-3, 140-5p and miR-143) [93]. These findings regarding miR-222, miR-142-3 and 140-5p are in accordance with findings in adults, in whom increased levels of these miRNAs have been correlated with an increased BMI and severe obesity [61]. Another study evaluated a complete miRNA panel in prepubertal children with obesity and found that various miRNAs were deregulated and related to BMI, adipose tissue percentage of body weight and other parameters of metabolic dysfunction [94]. One of them, miR-122, which was increased, correlated positively with dyslipidemia, NAFLD and insulin resistance in children [95], while several genes targeted by miR-122 have been associated with insulin resistance and skeletal muscle response to insulin. In adult NAFLD, miR-122 was significantly decreased in the liver but elevated in the circulation. It has been speculated that miR-122 is normally secreted by the liver to regulate hepatic function and cholesterol production, however, in obesity it is largely secreted by adipose tissue, perhaps in an attempt to maintain normal liver function [91]. Furthermore, obese rodents and humans, as well as children with NAFLD, have increased levels of miR-34a in vWAT, while miR-34a has been identified in adipose-derived exosomes and has been shown to inhibit M2 macrophage polarization promoting obesity-associated inflammation [93].

In a study by Thompson et al., obese children displayed a prominent increase in circulating miR-199a compared to lean controls [97]. Functionally, miR-199a is implicated in preadipocyte proliferation and differentiation and insulin signaling. In the same study, miR-21, miR-27b, miR-29a, miR-150 and miR-223 correlated significantly with BMI, especially miR-29a [97], already known to be associated with obesity and diabetes. miR-29 knockout mice were protected from diet-induced obesity and insulin resistance. Furthermore, in obese preschoolers three circulating miRNAs, miR-200c-3p, miR-190a and miR-95, correlated with insulin resistance [95]. Moreover, hsa-miR-125a-5p, hsa-miR-342-3p and hsa-miR-365b-3p could predict endothelial dysfunction in obese children aged 5–10 years [96]. Another miRNA shown to be correlated with obesity was miR-150; this miRNA mediates transformation between BAT and WAT. miR-150 knockout mice displayed body weight loss associated with hyperleptinemia and enhanced insulin sensitivity [98]. While miR-150 levels were increased in obese children, they were decreased in obese adults with diabetes mellitus compared to lean controls, suggesting differences in the profile of circulating miRNAs between children and adults that should be taken into account when evaluating potential biomarkers [97].

Exosomal lncRNAs in obesity. Most studies investigating exosomal lncRNAs have mainly evaluated the function of cancer cell secreted exosomal lncRNAs. Nevertheless, one study demonstrated that adipose tissue secretes exosomes containing the lncRNA HOTAIR, that subsequently communicate with intestinal cells and promote their proliferation, thus linking sedentary lifestyle and obesity with colorectal cancer [27]. Another study exploring the exosome content of adipose-derived stem cells (ASC) from lean and obese individuals, demonstrated that growth-arrest specific 5 (GAS5), large intergenic ncRNA (lincRNA)-VLDLR and metastasis associated lung adenocarcinoma transcript 1 (MALAT1) are increased in exosomes. Specifically, exosomal lincRNA-VLDLR secretion is increased in the omental fat of ASC derived from obese individuals, while exosomal MALAT1 expression is increased in subcutaneous ASC from lean individuals [99].

Circulating lncRNAs in obesity. There is mounting evidence that lncRNAs could provide insight into obesity-related gene regulation. In a recent study, 249 lncRNAs and 392 mRNAs were abnormally expressed in obese adults compared to controls with a normal BMI [100]. In particular, lncRNA-p5549, lncRNAp21015 and lncRNA-p19461 were comparatively decreased in obese subjects and significantly correlated with BMI, waist circumference, and waist-to-hip ratio, but not with obesity-related inflammatory biomarkers [100]. After 12 weeks of dietary intervention and body weight loss, circulating levels of lncRNA-p19461 increased and correlated negatively with insulin resistance [100]. Finally, the evaluation of lncRNA-mRNA interaction networks revealed that the Toll-like receptor (TLR) signaling pathway, a well-established pro-inflammatory pathway, and fatty acid metabolism had a strong interaction, probably influencing obesity risk [100].

Apart from genetic predisposition to obesity associated with functional polymorphisms affecting LEP and LEPR gene expression, epigenetic alterations in the transcriptional LEP gene regulation could lead to obesity by decreasing leptin secretion by adipocytes, suggesting that individuals with obesity and low leptin levels could remain sensitive to leptin and benefit from leptin therapy. Accordingly, Dallner et al. identified lncOb, that appears to increase LEP gene transcription by stabilizing the promoter complex loop; conversely, lncOb expression defects led to a form of obesity characterized by low leptin levels, that responded to leptin therapy [101]. In line with this finding, single-nucleotide polymorphisms (SNPs) in the lncOb region were identified in individuals with obesity and low leptin levels [102]. In another study, Lo et al. identified a lncRNA ~20kb upstream the LEP gene, which they named lnc-Leptin, whose expression was elevated in obesity, decreased by fasting, and induced by insulin, but which displayed a positive correlation with leptin expression in a spectrum of pathophysiological conditions, suggesting that this lncRNA acts probably as an enhancer-lncRNA [103].

Despite the fact that numerous lncRNAs have been identified as key regulators of adipocyte biology, the understanding of their role in childhood and adolescent obesity remains limited. A recent genome-wide association study (GWAS) analysis revealed an SNP in the lncRNA rhabdomyosarcoma 2-associated transcript (RMST) in children with severe obesity [104]. Furthermore, 1268 lncRNAs were shown to be differentially expressed in the adipose tissue of obese and non-obese children (531 increased and 737 decreased), as well as 1085 mRNAs (618 increased and 467 decreased) [105]. Gene ontology (GO) and pathway analysis of related genes demonstrated that 10 lncRNAs play a role in multiple biological pathways, including those implicated in immune and inflammatory reactions, fatty acid biosynthesis, osteoclast differentiation, and the AMPK signaling pathway [105]. Using quantitative Reverse Transcription polymerase chain reaction (qRT-PCR), lncRNA RP11-20G13.3, LINC00968, and AC011891.5 had comparatively increased expression levels, while expression of GYG2P1, RP11-529H2.1 and oligodendrocyte maturation–associated long intergenic nc RNA (OLMALINC) was comparatively decreased in children with obesity [105]. Expression levels of lncRNA RP11-20G13.3 demonstrated a positive correlation with BMI, waist circumference, waist-to-hip ratio, low-density lipoprotein (LDL) cholesterol, fasting insulin, high-sensitivity C-reactive protein (hsCRP) and leptin, and suppression of preadipocyte to adipocyte differentiation [105]. On the contrary, lncRNA GYG2P1 expression levels displayed a negative correlation with parameters of metabolic health, such as BMI, waist circumference, fasting insulin and triglycerides. These findings suggest that these two lncRNAs could be pivotal in the pathogenesis of childhood obesity [105].

In a study by Chen et al., lncRNA HLA complex P5 (HCP5) and LINC00839 were considerably increased in obese children compared to controls [106]. HCP5 polymorphisms have been associated with autoimmune disorders, leading to the hypothesis that HCP5-related disruption of immune response can contribute to childhood obesity; HCP5 functions as a competing endogenous RNA (ceRNA) binding to miR17-5p and increasing Ras-related protein R-Ras (RRAS) protein expression in the MAPK signaling pathway, which regulates adipocyte carbohydrate metabolism, thus, promoting childhood obesity development [106]. Respectively, HCP5 can act as a ceRNA binding to miR-27a/b to determine differentiation of adipocytes in childhood obesity, through Nemo-Like Kinase (NLK) in MAPK and Forkhead box O (FoxO) signaling pathways [106].

### 4.4. Clinical Use of Exosomes and Therapeutic Aspects

Exosomes represent a novel promising class of microvesicles with potential applications as biomarkers for various diseases and as therapeutic biomolecule carriers [11]. The ability of different cell types to display unique exosomal cargo, as well as the presence of exosomes in multiple biological fluids, can be exploited in the development of accurate non-invasive biomarkers for various conditions. For example, urinary exosomal miR-424 and miR-218 have been evaluated as biomarkers for Type 1 Diabetes Mellitus (T1DM) in children [107]. Compared to traditional biomarkers, exosomes are less complicated than the overall bodily fluids and have high content stability, facilitating long-term storage [12]. In addition, circulating miRNAs may be detected in the serum earlier during disease progression than many traditionally used protein biomarkers [97]. Obesity-associated circulating miRNAs, owing to their ability to reveal dynamically the current state of their originating cells, as well as to their efficient protection inside exosomes, constitute promising non-invasive biomarkers of obesity and related metabolic complications.

The establishment of circulating miRNAs as biomarkers of childhood obesity could provide a tool for evaluating the degree of obesity and risk factors for metabolic syndrome and T2DM prediction, which is of great importance for personalizing medical practice and preventing the future consequences of childhood obesity and associated comorbidities. For example, it has been suggested that miR-122 could potentially apply as a future biomarker for evaluating disease severity and monitoring disease progression in pediatric NAFLD [108]. Such an application could limit the need for invasive liver biopsies, usually required to diagnose this disease. Furthermore, circulating miRNAs could be used to monitor body weight-loss attempts, such as dietary restriction, physical programs and bariatric operations, all of which have been associated with up- or down-regulation of specific miRNAs. Lately, lncRNAs have also been attracting interest as prospective biomarkers for various disorders, especially cancer [109].

Regarding the use of exosomes as therapeutic biomaterials, naturally derived exosomes may bear therapeutic properties proportionate to the ones of their cell of origin or could be used as active biomolecule carriers [110], with potential applications in cancer, cardiovascular, neurologic, and autoimmune diseases. For instance, in T1DM, stem-cell derived exosomes display a protective action on beta-cell autoimmune destruction, while transplanted bone marrow exosomal miR-106b and miR-222 have been associated with enhanced b-cell proliferation and improvement of hyperglycemia [111]. Exosomes are coming to light as therapeutic delivery systems for miRNA mimetics or anti-miRNA oligonucleotides, because of their comparably high biocompatibility and low immunogenicity, as well as their increased capacity to carry nucleic acids and to target specific cells through their surface proteins. Various methods can be used to load miRNAs into exosomes, such as electroporation, active packaging by using proteins or conserved sequences of exosome enriched RNAs (eeRNAs) and transfection of isolated exosomes or of the parental cells to produce “hybrid” exosomes [112]. The above information suggests that modifying exosomal miRNA cargo could provide various health-related benefits and upgrade traditional therapeutic approaches. However, more studies are required to assess the feasibility of these applications and great attention should be paid regarding their possible adverse effects, as exosomes mediate various physiological procedures, that could possibly be disrupted if exosome cargo and function were altered [110]. Moreover, massive production of synthetic exosome analogues could provide a promising new method for drug delivery.

During the last decade, miRNAs have become promising therapeutic agents for many disorders such as metabolic syndrome, neurological and autoimmune disorders, and cancer [4,18]. More specifically, in childhood obesity, the attractive perspective of ameliorating insulin resistance and obesity-related inflammation, and of promoting browning of WAT could be accomplished by the activation or blockade of key circulating miRNAs. Recent studies have identified distinct circulating miRNA profile in non-syndromic obesity and genetic obesity types, such as Prader-Willi syndrome, associated with distinct molecular pathogenesis [113]. Further investigation could reveal specific miRNAs for therapeutic targeting of these rare disorders. Intriguingly, recently a group of miRNAs has been patented for the treatment of diseases associated with primary ciliopathies, including the genetic obesity syndrome Bardet-Biedl [114].

Another promising therapeutic strategy would be to target exosomal lncRNAs. This perspective has been evaluated mainly in cancer; however, obesity could be next. Pharmacological compounds could target lncRNAs that regulate white adipocyte differentiation, exploiting the recently uncovered structure and function of many lncRNAs. Alternative strategies could include stimulation of brown adipocytes by lncRNAs that possess such properties, such as Blnc1, or regulation of combined lncRNAs-miRNAs that interact during adipocyte differentiation, such as NEAT1 and miR-140 [72]. The establishment of lncRNAs as therapeutic targets remains a remote but promising perspective at this time.

## 5. Conclusions and Future Perspectives

Adipose tissue releases exosomes with distinct cargoes that constitute a novel way of metabolic organ inter-communication. These exosomes and their cargoes are altered in obesity and accompanying disorders, rendering them potential biomarkers for diagnosis and monitoring, as well as prospective therapeutic targets. Research on exosomes and metabolism keeps expanding and adipose-derived exosomes have been established as crucial components of inter-cellular communication networks, however, we are currently only beginning to unveil the complex mechanisms of exosome functions in adipose tissue and obesity pathogenesis.

Some challenges encountered include technical difficulties in exosome isolation, quantification, characterization of the cell of origin, as well as a high heterogeneity between findings, possibly attributable to sample differences and different isolation techniques. It is speculated that a combination of circulating miRNAs, rather than a single miRNA, orchestrate metabolic processes, setting the objective of elaborating a full profile of circulating miRNAs and elucidating the mechanisms of their involvement in obesity. Furthermore, as already mentioned, studies evaluating exosomal, especially adipose-derived exosomal miRNAs and lncRNAs, are rare, as most studies investigate circulating mRNAs and lncRNAs in blood samples, pointing out the need to identify the tissue origin of circulating miRNAs and lncRNAs in the future, and for further conducting studies regarding exosomal miRNAs and lncRNAs.

The roles of exosomes in obesity have been more extensively studied in adults than in children and adolescents. Therefore, a larger number of studies with more participants is required to draw safe conclusions regarding their participation and importance in childhood and adolescent obesity. We suggest that studies in the young age group could provide remarkable findings that will deepen our understanding of obesity, as children and adolescents have fewer comorbidities that could overshadow the mechanisms involved in obesity pathogenesis and progression.
